# Impact of Bronchoscopic Lung Volume Reduction with Valves on the Pulmonary Gas Exchange

**DOI:** 10.3390/jcm13082354

**Published:** 2024-04-18

**Authors:** Jane Winantea, Katharina Stiehl, Ruediger Karpf-Wissel, Faustina Funke, Hubertus Hautzel, Birte Schwarz, Heinz Steveling, Christian Taube, Filiz Oezkan, Kaid Darwiche

**Affiliations:** 1 Department of Pulmonology, Section of Interventional Pulmonology, University Medicine Essen, Ruhrlandklinik, University Duisburg-Essen, 45239 Essen, Germanykaid.darwiche@rlk.uk-essen.de (K.D.); 2 Department of Nuclear Medicine, University Hospital Essen, University Duisburg-Essen, 45147 Essen, Germany; 3 Department of Pulmonology, University Medicine Essen, Ruhrlandklinik, University Duisburg-Essen, 45239 Essen, Germany

**Keywords:** bronchoscopic lung volume reduction, endoscopic valves, lung emphysema

## Abstract

**Introduction:** Bronchoscopic lung volume reduction (BLVR) with endobronchial valves has been shown to be a safe and effective treatment for patients with severe lung emphysema. Previous studies have reported a benefit in pulmonary function, exercise capacity, and quality of life after BLVR-treatment. The effect of BLVR with valves on the pulmonary gas exchange and its association with clinical outcomes has not been analyzed to date. The primary goal of this study was to investigate the impact of BLVR on the pulmonary gas exchange and the impact of the target lobe selection in patients with discordant target lobes in high-resolution computed tomography (HRCT) scan and perfusion scan on the pulmonary gas exchange and clinical outcomes. **Methods:** In this single-center study, we retrospectively analyzed pulmonary function tests, 6-min-walk-tests, HRCT scans, perfusion scans, and blood gas analyses in 77 patients over the course of 6 months following BLVR treatment. **Results:** We observed that complete lobar occlusion with bronchoscopic valves leads to a transient impairment of pulmonary gas exchange. Despite this, an overall positive clinical outcome could be shown in patients treated with endobronchial valves. If the target lobe selection based on HRCT and perfusion scans is discrepant, a selection based on the HRCT scan tends to be associated with a better outcome than a selection based on the perfusion scan. **Conclusions:** Complete lobar occlusion with bronchoscopic valves leads to a transient impairment of pulmonary gas exchange but nevertheless results in an overall positive clinical outcome.

## 1. Introduction

Chronic obstructive pulmonary disease (COPD) is one of the most common chronic respiratory diseases, affecting 251 million people globally. It is estimated to become the third leading cause of death in 2030 by the World Health Organisation (WHO) [1]. The vast majority of COPD patients with predominant emphysema phenotype suffer from breathlessness due to hyperinflation, leading to notable impairment in exercise capacity and quality of life, despite optimal pharmacological therapy and rehabilitation [2]. The National Emphysema Treatment Trial (NETT) published in 2003 showed that patients with severe emphysema who underwent lung volume reduction surgery benefitted significantly with improvements in pulmonary function, exercise capacity, and quality of life [3]. Providing a less invasive option for patients who might not be suitable for lung volume reduction surgery, bronchoscopic lung volume reduction with endobronchial valves was introduced in Europe in the mid-to-late 2000s. Since then, significant clinical trials and studies further established the role of endobronchial valves. Patients with severe impairment of the pulmonary function (forced expiration in one second less than 40% of the predicted value) and pronounced hyperinflation (residual volume greater than 175% of the predicted value) were shown to benefit significantly from the treatment [4,5,6,7,8]. Based on the results of clinical trials, including the LIBERATE study, endobronchial valve treatment was approved by the Food and Drug Administration for use in the North American market in June 2018 [6].

**Figure 1:**
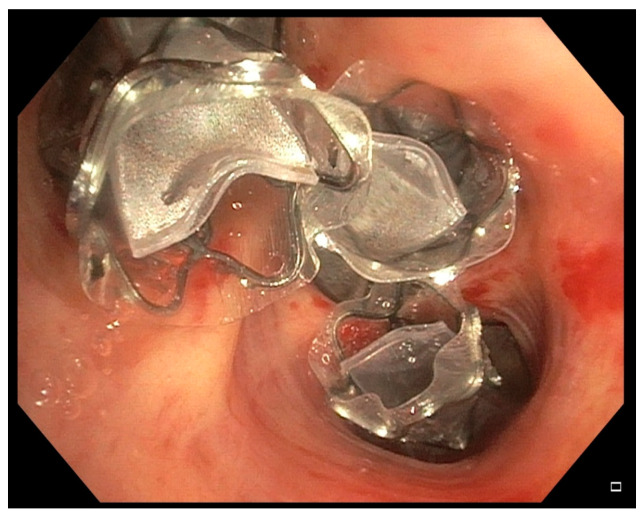
Bronchoscopic lung volume reduction of the left upper lobe. One-way emphysema valves (Zephyr, PulmonX, Redwood City, CA, USA) blocking the left upper lobe are shown. One valve is placed in LB 1/2, one valve in LB 3, and one valve in the lingula.

Bronchoscopic lung volume reduction (BLVR) with valves aims to reduce hyperinflation by blocking the most diseased lobe of the lung with one-way valves (Figure 1).

The treatment is reversible, and has been shown as a safe and effective therapy with clinically meaningful improvement in pulmonary function, exercise capacity, and quality of life [4,5]. BLVR has been proven to be an effective treatment option both in heterogeneous and homogeneous emphysema [6,7].

The absence of interlobar collateral ventilation and complete lobar occlusion, resulting in significant volume reduction of the target lobe, have been shown as an important predictor for the clinical response [4,8,9]. While previous studies have analyzed the effect of complete lobar occlusion based on pulmonary function and exercise capacity, it has not yet been examined to what extent the lobar occlusion influences the pulmonary gas exchange. We hypothesized that the pulmonary gas exchange would be affected by the lobar occlusion. As a widely practiced standard, the selection of the target lobe is based on the emphysema distribution in high-resolution computed tomography scan (HRCT) and perfusion scintigraphy [10]. However, in some cases, the most diseased lobe in HRCT and perfusion scintigraphy are not identical. In such cases, it is still unclear whether the selection of the target lobe, based on HRCT or perfusion scintigraphy, results in the best outcome. 

The primary goal of this study was to investigate the impact of BLVR on the pulmonary gas exchange, and the impact of the target lobe selection based either on HRCT or a perfusion scan on the pulmonary gas exchange and clinical outcome. 

## 2. Materials and Methods

To test the hypothesis of this study, we analyzed clinical data of patients with severe lung emphysema who underwent bronchoscopic lung volume reduction with one-way valves in our tertiary care center at Ruhrlandklinik Essen, Germany, retrospectively. The study complies with the Declaration of Helsinki, and the study protocol was approved by the local ethics committee of the University Duisburg-Essen (approval no. 15-6452-BO). 

### 2.1. Patient Selection and Valve Implantation

Patients treated with endobronchial valves in our center between 2015 and 2017 were included in this analysis. Patients, who underwent valve removal, revision, or replacement in the 6-month follow-up (FU) period were excluded. Patients with significant hyperinflation (residual volume RV > 200% pred.) and reduced forced expiration in one second (FEV1 ≤ 40% pred.) in their pre-treatment lung function test were evaluated for BLVR. A 6 min walk test (6-MWT), HRCT, as well as a perfusion scan were performed. Patient and target lobe selection for treatment with endobronchial valves was discussed prior to the treatment in a multidisciplinary emphysema board at our center in consideration of surgical and other endoscopic lung volume reduction techniques.

Moreover, fissure integrity was visually rated by radiologists experienced in HRCT evaluation. Absence of collateral ventilation was confirmed using catheter-based Chartis^®^ evaluation, as described previously [9].

Endoscopic valve implantation was performed in a standard manner described in previous studies [11,12]. In brief, all patients received a complete lobar occlusion of the most emphysematous lobe by implantation of one or several endobronchial valves (Zephyr^®^, Pulmonx, Inc., Palo Alto, CA, USA) under total intravenous anesthesia. All patients were monitored on the intensive care unit for 24 h. Chest radiography was performed 2 h post-intervention and on the next day to rule out pneumothorax and assess target lobe volume reduction (TLVR). To verify the correct position of the endobrochial valves and rule out valve dislocation, a bronchoscopy was performed two days after valve placement, as well as in the Fus after one month, three, six, and twelve months.

### 2.2. Clinical and Radiological Data Acquisition

Pulmonary function tests (PFTs) and 6-MWTs were evaluated one month, three months, and six months after the intervention. Pulmonary gas exchange was measured using the alveolar-arterial oxygen gradient (AaDO2) in a blood gas analysis at rest. Additionally, we evaluated oxygen desaturation (ΔSpO2) in patients with an identical rate of supplemental oxygen during a 6 min walk test before and after the BLVR intervention. Oxygen saturation was recorded continuously during the test. ΔSpO2 was calculated by subtracting the lowest oxygen saturation (SpO2) from the SpO2 at rest.

### 2.3. Selection of the Target Lobe

Even though the data was analyzed retrospectively, two interventional pulmonologists (CT1 und CT2) received only the CT scan and not the perfusion scan, while another two physicians specialized in nuclear medicine received only the lung perfusion scan and not the CT scan. All four physicians did not have access to other clinical information. Each of them was instructed to choose the most applicable target lobe, based on a visual quantification of the emphysema severity in each lobe. In the same manner, an interventional pulmonologist with a year-long experience in perfusion scan analysis and a nuclear medicine physician (PS1 and PS2) chose the target lobe based on the perfusion scan. 

TLVR was assessed based on chest radiography one day, three months, and six months after valve placement, as well as using HRCT 3 months post-intervention. Patients with complete lobar atelectasis and partial atelectasis were assigned to the TLVR group, whereas the non-TLVR group consisted of patients with no volume reduction or dystelectasis with negligible volume reduction. 

### 2.4. Statistical Analysis

Data were statistically analyzed using SPSS Statistics (IBM SPSS Statistics for Windows, Version 25). Baseline characteristics are presented as mean values and standard deviation. Paired samples student *t*-test was used to evaluate changes from baseline to post-intervention values. Not-normally distributed parameters, due to small sample size, were analyzed via Wilcoxon signed-rank-test. *p*-values < 0.05 were considered statistically significant. 

## 3. Results

### 3.1. Patient Characteristics

A total of 77 patients (mean age 62 years, 32 male) were included in the analysis. Baseline data are shown in detail in Table 1. Forty-three patients were on long-term oxygen therapy at baseline. In 25 (32.5%) patients, the left upper lobe was treated; in 23 (29.9%) the left lower lobe was treated; in 1 (1.3%) patient, the right middle lobe was treated; in 16 (20.8%) patients, the right upper lobe was treated; and in 11 (14.3%) patients, the right lower lobe was treated. One patient (1.3%) received valve treatment in both the right upper lobe and middle lobe.

### 3.2. Selected Target Lobe Based on HRCT and Perfusion Scan

The selected target lobe by CT1 and CT2 matched in all cases. The target lobes selected in the HRCT scans did not match the treated lobe in 7 (9%) of the cases. Selection of the target lobes using PS1 and PS2 did not match in three of the cases, corresponding to an inter-observer agreement of 96.1%. In the event of disagreement upon the target lobe, a third interventional pulmonologist (PS3) was queried to choose a target lobe. The selection of PS3 matched those of PS1 in two cases and PS2 in one case. Overall, the target lobes selected based on the perfusion scan did not match the treated lobe in eight (10%) cases. 

### 3.3. Primary Outcome Measures

#### Pulmonary Gas Exchange

**Figure 2:**
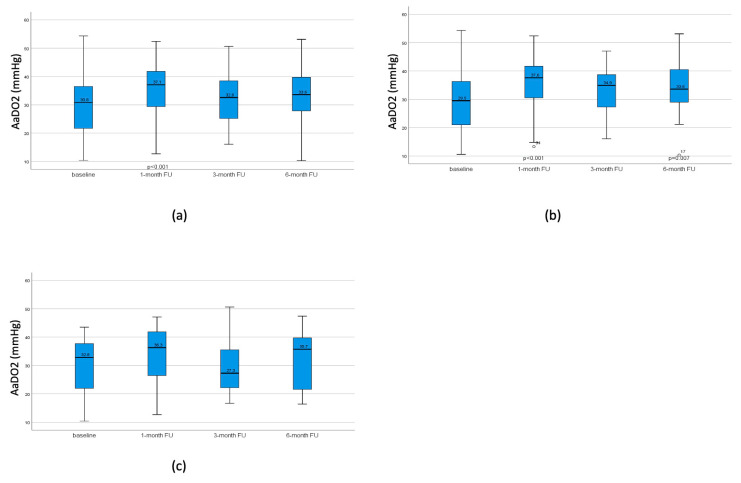
AaDO2 at baseline, after 1 month, 3 months, and 6 months. (**a**) AaDO2 in the all patients at baseline, after 1 month, 3 months, and 6 months, showing a significant worsening of the gas exchange in the one-month-follow up, returning to baseline after 3 months. (**b**) AaDO2 in patients with TLVR, at baseline, after 1 month, 3 months, and 6 months, showing a significant worsening of the gas exchange in the 1-month-follow up and 6 month-follow-up. (**c**) AaDO2 in patients who did not achieve TLVR; no significant change was observed after 1 month, 3 months, and 6 months follow-up. *p*-values compared to baseline. No *p*-value indicates non-significance.

A significantly worsened pulmonary gas exchange was observed after one month in both the whole group and the TLVR group, shown in Figure 2. However, the pulmonary gas exchange showed no relevant impairment after three and six months. Using the same rate of supplemental oxygen in 24/77 patients, significantly lower desaturation rates were detected in the 6 months-FU (∆SpO2% from 6.71(±4.8)% to 4.96(±3.2)%, *p* = 0.026).

### 3.4. Secondary Outcome Measures

#### 3.4.1. Target Lobe Volume Reduction

A significant lung volume reduction was achieved in 48 patients (62%), whereas 29 patients (38%) remained without TLVR. Significant lung volume reduction persisted in 87.5% of cases, while six patients lost their initial lung volume reduction in the 6-month FU. 

#### 3.4.2. Pulmonary Function Test and Exercise Capacity

**Figure 3:**
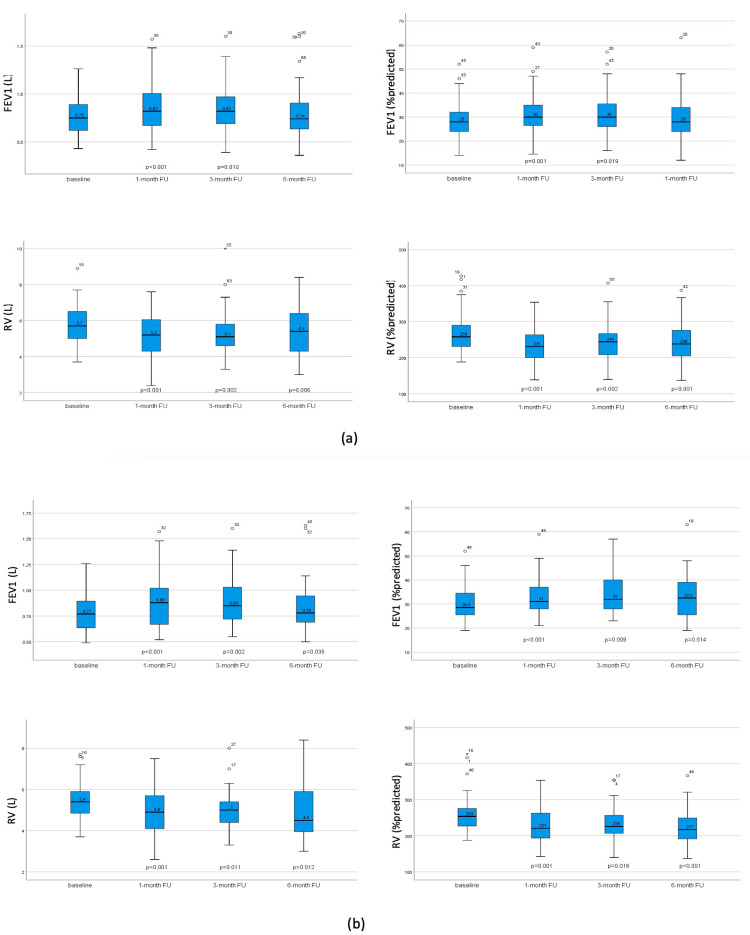

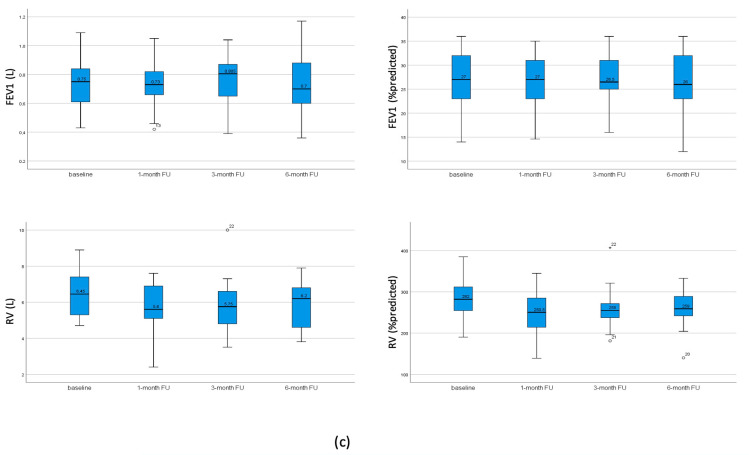
FEV1 and RV at baseline and after 1 month, 3 months, and 6 months post-intervention. (**a**) FEV1 and RV in all patients with significant improvement of FEV1 and reduction of hyperinflation. (**b**) FEV1 and RV in patients with TLVR, showing significant improvement of FEV1 and reduction of hyperinflation in all follow ups. (**c**) FEV1 and RV in patients without TLVR, with no significant change. *p*-values compared to baseline. No *p*-value indicates non-significance.

FEV1 and RV at baseline as well as after 1 month, 3 months, and 6 months post-intervention are presented in Figure 3. In the whole sample group, FEV1 increased significantly one month post-intervention, with the improvement maintained after three months. Patients with TLVR improved in all three FU both in terms of FEV1 and hyperinflation, whereas patients without atelectasis displayed no significant changes after valve implantation. Exercise tolerance measured using 6-MWT significantly improved in the whole group after 1 and 3 months.

**Figure 4:**
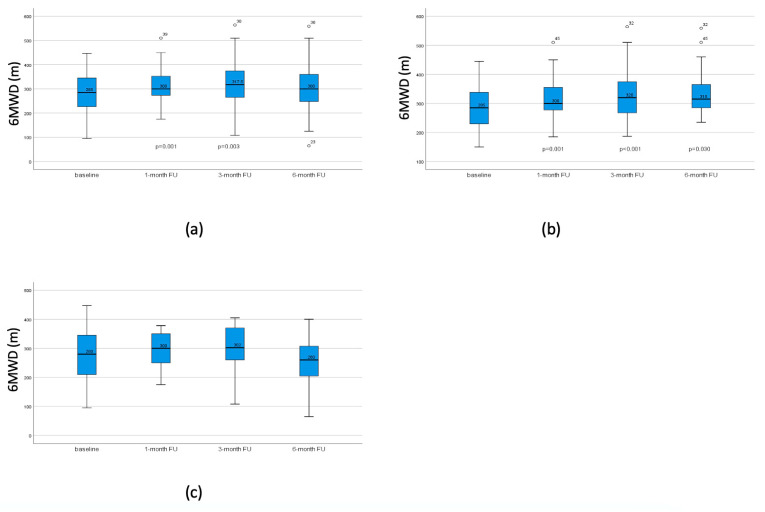
6-MWD at baseline, after 1 month, 3 months, and 6 months. (**a**) 6-MWD in all patients with significant improvement after 1 month, returning to baseline after 3 months. (**b**) 6-MWD in patients with TLVR, showing an improvement in the one-month-follow up and six-month-follow up. (**c**) 6-MWD in patients without TLVR, showing no significant change in all follow ups. *p*-values compared to baseline. No *p*-value indicates non-significance.

In patients with TLVR, exercise capacity significantly improved in all three FUs. In patients without successful lobar occlusion, walking distances did not significantly improve (Figure 4).

#### 3.4.3. Impact of Discrepancy of the Target Lobe in HRCT and Perfusion Scan on Clinical Outcome

**Figure 5:**
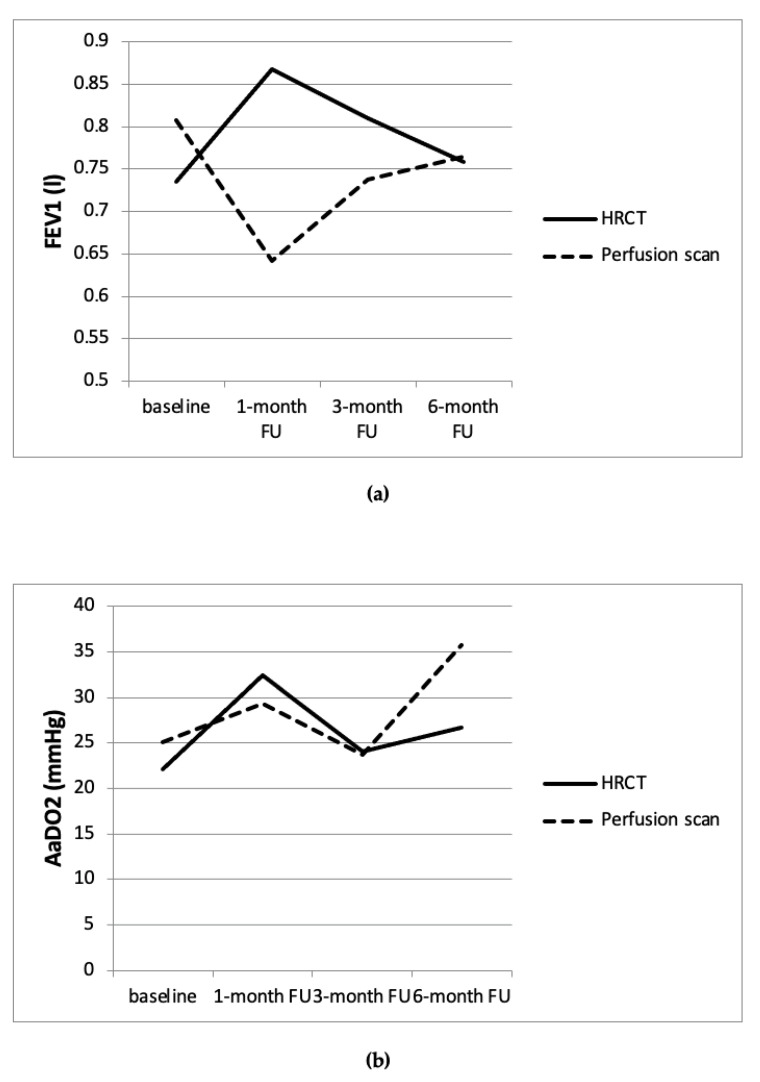
FEV1 and AaDO2 in patients with discrepancy in the target lobe. (**a**) FEV1 in patients with discrepancy in the target lobe, showing an improvement of FEV1 in patients, where the target lobe matches the HRCT and a worsening of FEV1 in patients, where the target lobe was selected based on the perfusion scan. (**b**) Gas exchange in patients with discrepancy in the target lobe, showing a more distinct worsening of the AaDO2 in patients, where the target lobe matches the HRCT in the one-month-follow up, evening out in the next follow ups.

A relevant change in the clinical outcome in patients with a discrepant target lobe based on either HRCT or perfusion scan could be shown. We could demonstrate a relevant increase of FEV1 from baseline after one month, where the treated lobe matched the one selected based on the HRCT (*p* < 0.05). On the other hand, although statistically not significant, in patients in which the treated lobe matched the lobe selected in the perfusion scan but not the HRCT, FEV1 decreased by 0.13 L after one month (*p* = 0.076). Furthermore, in patients where the treated lobe matched the selection based on the perfusion scan but not the HRCT, no significant change in the AaDO2 was observed, whereas in patients where the treated lobe matched the one based on the HRCT but not the perfusion scan, the gas exchange responded exactly like in the whole group, initially worsening significantly, then evening out in the further follow-ups (Figure 5).

## 4. Discussion

We hypothesize that excluding the treated lobe from ventilation affects the overall gas exchange. In this study, we can show a significant worsening of the pulmonary gas exchange one month after bronchoscopic valve treatment. The worsening of the pulmonary gas exchange can be explained by a ventilation perfusion mismatch as a result of the lobar collapse in the treated lobe. However, the pulmonary gas exchange returned to baseline after 3 months. The best possible explanation for this is a delay in the reduction of perfusion in the volume-reduced lobe. Additionally, we showed that the worsening of the pulmonary gas exchange 4 weeks after treatment did not co-concur with a worsened exercise capacity measured using 6-MWT. Moreover, a decrease of desaturation despite an identical rate of supplemental oxygen during the 6-MWT was observed after 6 months, presumably because the reduction of pulmonary hyperinflation compensates for the impaired gas exchange. This corresponds to the finding of Pizarro et al. and Chung et al., showing a redistribution of the ventilation and perfusion to the untreated lobe after completed lobar occlusion with valves [13,14,15]. 

Our finding implicates that, while the worsening of the gas exchange diminishes, patients benefit further from the reduction of hyperinflation, which has been shown to be associated with an improvement of both breathing mechanics and cardiac function [16,17,18]. These findings are in accordance with the fact that patients with homogenous emphysema also benefit from valve treatment, despite a relatively lower destruction degree of the target lobe [7]. We assume that the higher the degree of hyperinflation, the less clinically significant the impairment of the gas exchange. This could contribute to patient selection for valve treatment, especially patients needing high amounts of supplemental oxygen before treatment. In these patients, valve treatment can be considered despite risk of transient increase of supplemental oxygen if a pronounced hyperinflation is present. In any case, informing patients about a possible transient worsening of the gas exchange, or even a temporary need of supplemental oxygen, should be considered as part of the informed consent.

It is conceivable that the transient deterioration of the pulmonary gas exchange is correlated to the degree of destruction of the treated lobe. As quantitative HRCT analysis were not yet consistently available in our center at the time of the valve placement, we were not able to examine whether the change in pulmonary gas exchange correlated with the level of destruction and/or volume of the treated lobe. This therefore remains unanswered and subject to further study. 

The subsequent selection of the target lobe by experts, visually either based on HRCT or a perfusion scan, blinded to the actual treated lobe, showed a discrepancy in a few cases. In these cases, we could show that clinical outcome seems to be better if the target-lobe is selected based on HRCT scan. Also, the fact that visual selection of the target lobe based on a perfusion scan was in some cases inconsistent between two experts indicates that this may possibly be less reliable than the HRCT. Thomsen et al. showed that the perfusion of the target lobe did not have any effect on the clinical outcomes, which supports our hypothesis [19]. A possible explanation could be the lack of single photon emission computed tomography (SPECT) imaging with lobe-based quantification for the selection of the target lobe. 

Apart from the retrospective nature of the study, there are some further limitations that should be discussed. Patients in our study showed slightly less improvement in clinical outcomes as compared to those observed in lately published randomized clinical trials [6,7,20,21]. This may be accounted for by lower percentage of patients achieving a TLVR compared to other trials. We speculate that this is explained by the lack of CT-scan based quantitative analysis of the fissure completeness before treatment at that time in our center. 

As few FU data are missing, it is possible that patients who had a greater benefit after treatment did not keep their FU appointment as regularly and reliably as patients who responded less to the treatment or had exacerbations. Hence FU data could be biased, and may under-represent patients with better clinical outcomes. Additionally, pulmonary gas exchange was measured in AaDO2 calculated from capillary blood gas analysis. The retrospective character of the study carries the potential risk of not strictly standardized conditions for the collection of the blood gas analysis.

## 5. Conclusions

To the best of our knowledge, this is the first study to show that a complete lobar occlusion with bronchoscopic valves leads to a transient impairment of pulmonary gas exchange. Despite the impairment of pulmonary gas exchange, we observed an overall positive clinical outcome of bronchoscopic lung volume reduction, in line with previous publications on this topic. If the target lobe selection based on HRCT and perfusion scans is discrepant, a selection based on the HRCT scan tends to be associated with a better outcome than a selection based on the perfusion scan.

## Figures and Tables

**Table 1:** Patients characteristics at baseline.

Gender	32 males (41.6%) 45 females (58.4%)
Age (years)	62.36 ± 7.17
BMI (kg/m^2^)	22.79 ± 4.0
FEV1 (L)	0.77 ± 0.2
FEV1 (%predicted)	28.64 ± 6.6
IVC (L)	2.3 ± 0.8
IVC (%predicted)	66.2 ± 16.1
RV (L)	5.79 ± 1.08
RV (%predicted)	268.29 ± 53.03
KCO (%predicted)	32.8 ± 11.9
AaDO2 (mmHg)	29.09 ± 9.95
Patients on long-term oxygen therapy	43 (55.8%)
6-MWD (m)	286.86 ± 77.2

## Data Availability

The original contributions presented in the study are included in the article material, further inquiries can be directed to the corresponding authors.
